# Emerging challenges for mosquito-borne disease control and the promise of symbiont-based transmission-blocking strategies

**DOI:** 10.1371/journal.ppat.1013431

**Published:** 2025-08-22

**Authors:** Han Gao, Wenqian Hu, Chunlai Cui, Yiguan Wang, Yitong Zheng, Marcelo Jacobs-Lorena, Sibao Wang

**Affiliations:** 1 School of Basic Medical Sciences, Suzhou Medical College of Soochow University, Suzhou, China; 2 New Cornerstone Science Laboratory, Key Laboratory of Insect Developmental and Evolutionary Biology, State Key Laboratory of Plant Trait Design, CAS Center for Excellence in Molecular Plant Sciences, Shanghai Institute of Plant Physiology and Ecology, Chinese Academy of Sciences, Shanghai, China; 3 CAS Center for Excellence in Biotic Interactions, University of Chinese Academy of Sciences, Beijing, China; 4 Shanghai Institute of Wildlife Epidemics, School of Life Sciences, East China Normal University, Shanghai, China; 5 Department of Molecular Microbiology and Immunology, Malaria Research Institute, Johns Hopkins Bloomberg School of Public Health, Baltimore, Maryland, United States of America; National Institutes of Health, UNITED STATES OF AMERICA

## Abstract

Mosquitoes serve as vectors for a variety of pathogens that cause life-threatening diseases, such as malaria, dengue, Zika, and yellow fever. With the rise of antimalarial drug resistance and a lack of therapeutics or prophylactics for dengue and Zika, current disease control strategies rely heavily on mosquito population management. However, the effectiveness of conventional approaches is increasingly compromised, highlighting an urgent need for innovative tools to combat mosquito-borne diseases. One promising strategy for blocking the transmission of these diseases is to populate mosquitoes with anti-pathogen gut symbionts. Here, we discuss the major challenges facing current mosquito-borne disease control efforts and explore how mosquito gut microbiota-based control strategies may address them. We highlight recent advances that may accelerate field applications and offer perspectives on future directions and the translational potential of symbiont-based strategies for mitigating mosquito-borne disease transmission.

## 1. Introduction

**Fig 1:**
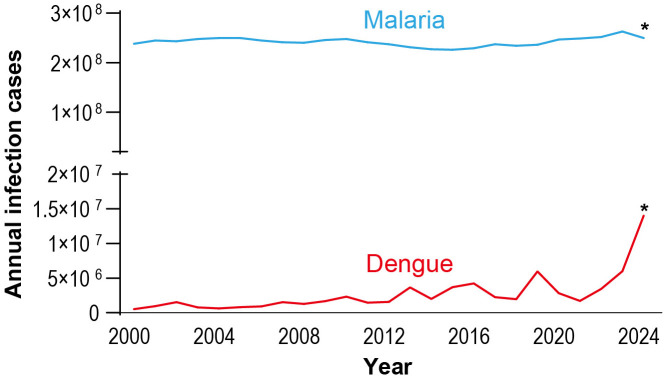
Annual malaria and dengue cases during the past two decades. Data adapted from WHO malaria report and dengue record [13,94]. The asterisks indicate estimated data (by website https://vizhub.healthdata.org/) as they are currently not available from WHO.

Mosquitoes are vectors of a wide range of pathogens responsible for major global diseases, including malaria, dengue, Zika, yellow fever, and lymphatic filariasis, threatening the health of over half the world’s population [1]. Traditional mosquito-borne disease (MBD) control strategies have primarily relied on vector management approaches, such as the use of chemical insecticides, environmental management, and protective measures like bed nets and repellents [2,3]. However, the effectiveness of these interventions is increasingly undermined by the rapid emergence of insecticide resistance [4,5], altered mosquito behaviors (e.g., outdoor or early biting) [6], and the global spread of invasive mosquito species [7,8]. As such, the incidence and geographic distribution of MBDs continue to expand (Fig 1), necessitating the urgent need for innovative, effective, and sustainable intervention strategies.

Current malaria control efforts, while currently effective in most regions, have recently stalled due to the emergence and spread of artemisinin-resistant *Plasmodium* parasites in Southeast Asia and Africa, which have complicated traditional treatment and intervention programs [9,10]. Although progress has been made in vaccine development (e.g., RTS,S and R21 candidate [11]) and next-generation antimalarial drugs (e.g., KAF156 and DSM265 [12]), their moderate efficacy, limited production capacity, and delivery hurdles mean they alone cannot secure elimination, underscoring the need for the introduction of innovative and integrated approaches to achieve long-term malaria elimination goals.

**Fig 2:**
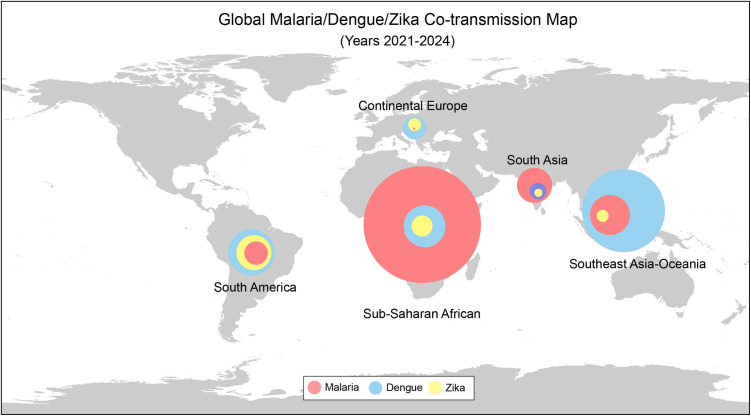
Global concurrent distribution map of malaria, dengue, and Zika diseases from years 2021 to 2024. Data adapted from WHO malaria report and dengue record. The world map was created using R package “rnaturalearth” (https://docs.ropensci.org/rnaturalearth/).

Meanwhile, the global burden of arbovirus diseases—including dengue, chikungunya, Zika, and West Nile virus—continues to rise, characterized by unprecedented case numbers and expanding geographical distributions [13–16]. Although vaccines such as Dengvaxia and Qdenga have been licensed for dengue control, their impact has been limited due to factors including suboptimal vaccine coverage, serotype-specific protection, and the risk of antibody-dependent enhancement [15,17]. In 2024, more than 14 million dengue cases and over 10,000 dengue-associated deaths were reported globally, exceeding all previous records [13]. Moreover, overlapping distributions of *Anopheles* and *Aedes* mosquitoes in many endemic regions facilitate concurrent transmission of multiple pathogens (Fig 2), leading to co-infections involving malaria, arboviruses, intestinal parasites, and other pathogenic microorganisms [18–22], further complicating disease diagnosis and treatment of MBDs. Furthermore, emerging pathogens such as *Plasmodium knowlesi* [23], Oropouche virus [24], and newly identified mosquito-associated *Rickettsia* species [25] further complicate the landscape of MBD control.

**Fig 3:**
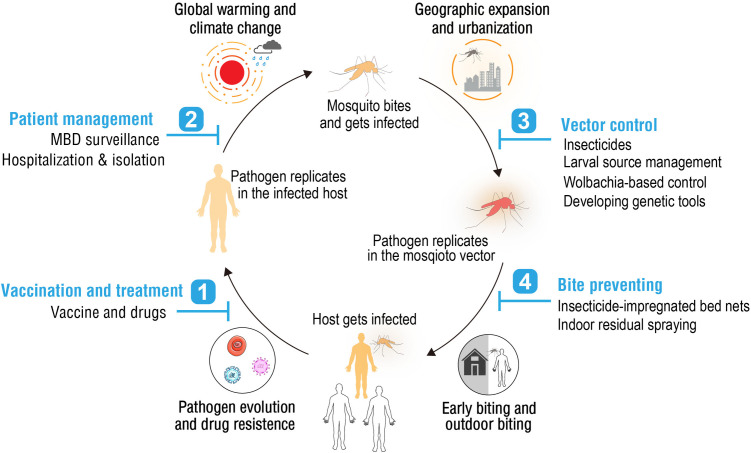
Emerging challenges to mosquito-borne disease (MBD) control. Present MBD control strategies (blue numbered) and emerging challenges that may reduce their effectiveness.

Limitations in MBD control are largely attributed to inadequate intervention measures, insufficient and unstable funding, and the emergence of new challenges to vector control efforts (Fig 3). A key contemporary challenge is the expansion of invasive mosquito species, fueled by global trade, urbanization, and climate change. For instance, modeling studies suggest that warming temperatures may facilitate the establishment of *Aedes aegypti* in southern Europe and the US Midwest by 2050 [26,27], while *Anopheles stephensi* has already begun spreading into urban areas of the Horn of Africa [28,29]. These emerging situations may exacerbate disease transmission and undermine existing control infrastructures [30–33]. Moreover, sustained vector control pressure has led to significant changes in mosquito behavior, reducing the efficacy of conventional indoor-targeted interventions such as insecticide-treated nets and indoor residual spraying [34,35]. For example, *Anopheles funestus* populations in East Africa have increasingly shifted to outdoor biting in the early evening [36], while *Ae. aegypti* in temperate regions has expanded its feeding activity to dawn and dusk hours [37,38]. Global warming and extreme climate events further complicate control by expanding mosquito habitats, enhancing pathogen replication rates, and disrupting established intervention programs [39,40]. Compounding these biological and environmental challenges, shifting national policy priorities and reduced donor commitment have led to inconsistent funding and diminished political will, threatening the continuity and coordination of global MBD control programs.

Recent efforts have increasingly focused on leveraging the mosquito microbiota, given its crucial role in shaping vector competence for pathogens such as *Plasmodium* and various arboviruses [41–46]. Symbiont-based transmission-blocking strategies, or paratransgenesis—where mosquitoes are colonized with either natural or genetically modified microbes that interfere with pathogen development—have emerged as promising, cost-effective tools for disease control [47–49]. Several recent reviews have summarized the progress of symbiont-based approaches [50–52], highlighted the microbial diversity in insect vectors [53–55], and examined the complex tripartite interactions among gut microbes, vectors, and pathogens [53,56–58]. While considerable progress has been made in laboratory and semi-field studies, further work is needed to optimize microbiota-based interventions for field-scale applications.

In this review, we focus specifically on strategies involving the manipulation of mosquito gut microbiota for the control of MBDs. We do not elaborate on *Wolbachia*- or *Microsporidium*-based approaches, as these have been discussed elsewhere [59–61]. Instead, we critically examine the promises and limitations of gut microbiota-based interventions, emphasizing recent advances, regulatory concerns, and practical considerations for achieving sustainable, large-scale implementation in endemic settings.

## 2. Conceptual shift

Population suppression of mosquitoes remains the main strategy to curb MBDs. Chemical insecticides are widely used for rapid response, especially during outbreaks. However, mosquitoes rapidly evolve multiple and complex resistance mechanisms that can outpace the development of new insecticidal compounds [62]. In addition, the overuse of insecticides raises concerns about off-target effects on beneficial insects, potential ecological imbalances, and risks to human health. These challenges underscore the urgency of transitioning from single‑mode chemical control to a diversified, biologically informed toolbox aimed at reducing mosquito populations or curbing their vector competence through more sustainable and ecologically compatible approaches.

### 2.1. Alternative strategies to suppress mosquito populations

In recent years, several strategies have been developed to suppress mosquito populations, including the release of *Wolbachia*-infected or irradiated sterile males [63], the use of entomopathogenic fungi [64], and genetically modified mosquitoes combined with gene-drive systems [65]. Among these, *Wolbachia*-based and sterile male release strategies have been implemented in countries such as Singapore, Mexico, and Brazil, showing strong efficacy in reducing MBD transmission [61]. Entomopathogenic fungus and gene-drive strategies have also shown promising results in laboratory and semi-field settings. However, each approach faces distinct challenges.

### 2.2. Reducing vector competence

**Fig 4:**
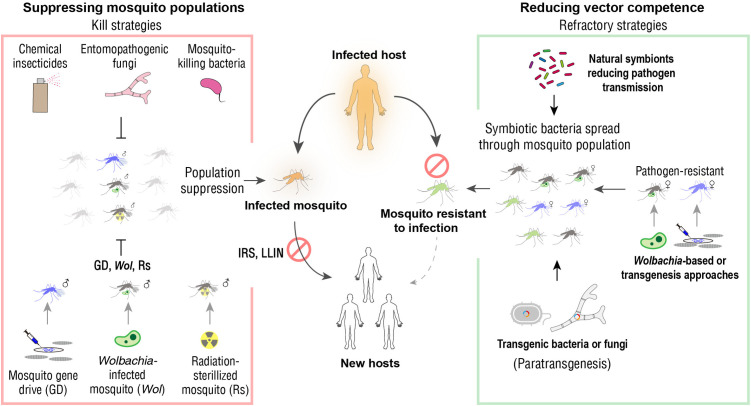
A summary of current MBD control concepts and strategies. Current MBD control strategies are based on two concepts: suppression of mosquito populations (kill strategy) and reduction of vector competence (refractory strategy). IRS: indoor residual spraying; LLIN: long-lasting insecticidal net.

Given the limitations of existing MBD control measures and the emergence of new challenges, there is growing interest in developing sustainable, versatile, ecologically friendly, and low-cost strategies. Efforts to eradicate mosquito populations have proven to be technically challenging and to raise ecological concerns. As a result, reducing vector competence—the ability of mosquitoes to transmit pathogens—has emerged as a more pragmatic and targeted approach (Fig 4). This paradigm shift aligns with the broader principles of the “One Health” framework, which emphasizes the interconnectedness of human, animal, and environmental health. Rather than seeking to eradicate mosquitoes, rendering them refractory to pathogen infection and thereby blocking disease transmission represents a sustainable strategy for MBD control. Heterogeneity in vector competence arises from a complex interplay among genetic background, microbiota composition, and environmental factors. Notably, the mosquito’s holometabolous life cycle sustains a dynamic and highly diverse microbial community, which contributes to digestion, nutrition, growth, fertility, and immune defense [66,67]. The intimate relationship between mosquitoes and their microbiota, together with conclusive evidence that microbial constituents strongly shape mosquito competence, underscores the potential for microbiota-driven approaches to MBD prevention.

## 3. Mosquito microbiota

Mosquito-associated microbes, whether residing in the gut lumen or as endosymbionts, can profoundly modulate vector competence by affecting pathogen replication and survival within the mosquito. Certain bacterial communities or strains can inhibit parasites or arboviruses by occupying binding sites or secreting antimicrobial factors, while also priming the mosquito’s innate immune pathways (such as Toll, IMD, and JAK/STAT) to curb infection. Notably, most of the mosquito microbiota resides in the lumen of the midgut, the site of the initial pathogen entry into the mosquito. This potential direct bacteria-pathogen interaction makes the midgut compartment a prime target for MBD intervention. Symbiotic control of MBDs has been under development for two decades and has shown much promise in the laboratory. Here we focus on the historical development of this strategy, highlighting recent progress of this approach, and the promises of these advances to address the challenges of MBD control. Mosquito gut bacterial strains that suppress pathogen development are listed in Table 1. As this review focuses specifically on gut bacteria-based strategies, Table 1 does not include intracellular *Wolbachia* [51,59–61], entomopathogenic fungi [64,68], *Microsporidium* [69], or insect-specific virus [70,71].

**Table 1:** Mosquito gut bacterial strains that suppress pathogens and published in the past two decades.

Mosquito species	Bacteria strain	Effectors	Function	Semi-field test	Refs
**Genetically modified bacteria**					
*An. stephensi*	*E. coli*	scFv	Inhibits *P. berghei*	*/*	[74]
*An. stephensi*	*E. coli*	SM1 and phospholipase-A(2)	Inhibits *P. berghei*	*/*	[95]
*An. gambiae*	*Pantoea agglomerans*	scorpine, EPIP, Shiva-1, mPLA2, Pro:EPIP	Inhibits *P. falciparum* and *P. berghei*	*/*	[47]
*An. stephensi*	*Serratia marcescens* AS1	MP2, scorpine, Shiva1, mPLA2, EPIP	Inhibits *P. falciparum*	*/*	[48]
*An. stephensi*	*Asaia*	scorpine	Inhibits *P. berghei* under bloodmeal promoter	*/*	[75]
*An. stephensi* *Ae. aegypti* *Ae. albopictus*	*Serratia marcescens* AS1	DN59, Z2 scorpine, Shiva-1	Inhibits DENV, ZIKV, *P. falciparum* and *P. berghei* under bloodmeal promoter	Semi-field big cage trial conducted in China	[76]
**Natural bacteria**					
*An. albimanus*	*Serratia marcescens, Enterobacter cloacae, Enterobacter amnigenus*	/	Inhibits *P. vivax* infection	*/*	[96]
*An. gambiae*	*Enterobacter* (*Esp_Z*)	Induce ROS production	Inhibits *P. falciparum*	*/*	[43]
*An. stephensi* *An. gambiae*	*Asaia* SF2.1	Activate mosquito immunity	Inhibits *P. berghei*	*/*	[97]
*An. stephensi*	*Serratia marcescens* HB3	*/*	Inhibits *P. berghei*	/	[82]
*An. stephensi* *An. gambiae*	*Serratia marcescens* Y1 and *Serratia ureilytica* Su_YN3	Activate mosquito immunity	Inhibits *P. berghei*	/	[41,98]
*An. stephensi* *An. gambiae*	*Serratia ureilytica* Su_YN1	Secreted lipase AmLip and outer membrane vesicles	Lysis *P. falciparum* and *P. berghei*	Ongoing semi-field trials conducted in Burkina Faso	[41]
*An. stephensi* *An. gambiae*	*Delftia tsuruhatensis* TC1	Secreted hydrophobic molecule harmane	Inhibits the development of female *Plasmodium* parasite gametes	Semi-field trials conducted in Burkina Faso	[44]
*Ae. albopictus* *Ae. aegypti*	*Rosenbergiella*_YN46	glucose dehydrogenase (RyGDH)	Inhibits DENV and ZIKV.	Semi-field greenhouse test conducted in China	[45]
*An. gambiae*	*Chromobacteriumsp. Panama* (Csp_P)	*/*	Kills adult mosquitoes	Enclosed field trials conducted in Burkina Faso	[99]

### 3.1. Paratransgenesis

One primary symbiotic control strategy, commonly referred to as paratransgenesis, uses genetically modified microbes to inhibit pathogen transmission by the vector. The concept was first introduced in the 1990s, when *Rhodococcus rhodnii* bacterium—a gut symbiont of the Chagas disease vector *Rhodnius prolixus*—was engineered to produce antimicrobial peptides that inhibit *Trypanosoma cruzi* development [72,73]. This approach was later adapted to mosquitoes using engineered *Escherichia coli*, *Asaia*, and *Pantoea* bacteria to express lytic peptides (scorpine, shiva1, etc.) targeting *Plasmodium* parasites, or peptides disrupting *Plasmodium* ookinete infection (SM1 and MP2, *etc.*), to block *Plasmodium* transmission by *Anopheles* mosquitoes [47,74,75]. Recent advances in this area include the use of *Serratia* strains that are vertically and horizontally transmitted, and can spread through mosquito populations [48]. Additionally, blood meal-inducible systems have been employed to drive the production of effector molecules in a temporally regulated manner, thereby minimizing potential impacts on mosquito and bacteria fitness [75,76]. Genetic engineering of entomopathogenic fungi, such as *Metarhizium anisopliae* and *Beauveria bassiana*, was used to combine *Plasmodium*-killing with mosquito-killing [77] or to enhance mosquito-killing efficacy [78].

Notably, no paratransgenic strategies have been specifically designed to target arboviruses so far, although such an approach has been shown to work in honey bees, where genetically modified *Apis mellifera* symbionts deliver RNAi constructs to reduce virus infection [79]. Current approaches tend to focus on a single pathogen, or closely related pathogens, in a particular vector, although some effectors may have broader pathogen blocking activity. For instance, scorpine and shiva1, two lytic peptides used in paratransgenic systems against *Plasmodium* parasites, may also target other eukaryotic pathogens such as *Wuchereria bancrofti* [80]. A recent work by our group explored polyvalent paratransgenic tools that simultaneously target multiple MBD pathogens, to address the concurrent transmission of malaria, dengue, and Zika. We engineered the symbiotic bacterium *Serratia* AS1, which efficiently spreads through both *Anopheles* and *Aedes* populations, to simultaneously produce anti-arbovirus and anti-*Plasmodium* effector molecules. Expression of these effectors is tightly regulated by a blood meal-inducible promoter, ensuring activation only in the presence of a blood meal. This selective and conditional expression strategy minimizes fitness costs to both the symbiont and the mosquito host, while also reducing potential off-target effects on non-vector organisms. Laboratory and contained semi-field experiments demonstrated that mosquitoes colonized with the engineered strain, AS1-TK, conferred strong refractoriness to *Plasmodium* and arbovirus infections in *Anopheles* and *Aedes* mosquitoes, respectively [76]. This study establishes a foundation for the use of pluripotent engineered symbiotic bacteria to combat the concurrent transmission of malaria and arbovirus diseases by vector mosquitoes.

### 3.2. Natural bacteria

Paratransgenic approaches, which utilize genetically engineered symbiotic bacteria to deliver anti-pathogen effectors, have shown great promise in laboratory setting. However, their translation to field application raises important biosafety and regulatory concerns, particularly when translating from laboratory studies to field implementation. As a result, increasing attention has been directed toward identifying naturally occurring symbiotic bacteria with inherent anti-pathogen activity as a potentially more acceptable and scalable alternative. Early studies showed that depletion of the gut microbiota from *Anopheles* mosquitoes often leads to increased *Plasmodium* parasite loads [81], implying that components of the native microbiome may exert a protective effect, partially through the priming of basal immune responses. Several bacterial strains have since been associated with reduced parasite burden. For instance, *Serratia marcescens* HB3 [82] and *Enterobacter* sp. Esp_Z [43] have both been shown to inhibit *Plasmodium* development in the mosquito midgut. Microsporidia MB, a naturally occurring symbiont in *Anopheles* mosquitoes that inhibits *Plasmodium* development, spreads through maternal inheritance and mating without harming mosquito fitness, offering the potential to curb malaria transmission [69]. More recently, epidemiological analyses or laboratory observations of mosquito populations exhibiting high *Plasmodium* resistance have led to the discovery of strains, such as *Serratia ureilytica* Su_YN1 (isolated from field-collected mosquitoes) [41] and *Delftia tsuruhatensis* TC1 (isolated from laboratory-reared mosquitoes [44]). In both cases, the anti*-Plasmodium* effectors were identified: Su_YN1 was found to secrete a lipase, while TC1 produces the small organic compound harmane. Notably, *Serratia* Su_YN1 can be abundant in the *Anopheles* mosquito gut and spreads effectively via both horizontal and vertical transmission (including transstadial passage). Furthermore, this *Serratia* outer membrane-mediated lipase delivery mechanism [83], and quorum sensing-based colonization strategy [46] have been elucidated. Such detailed insights into how natural bacterial strains inhibit mosquito-borne pathogens will be instrumental in advancing the refinement of symbiotic control approaches, thereby facilitating their deployment for malaria control and accelerating progress toward field implementation.

The influence of mosquito gut microbiota on flavivirus transmission is complex and context-dependent. It was shown that depletion of the gut microbiota from *Ae. aegypti* mosquitoes decrease DENV infectivity, leading to the identification of certain *Serratia* strains that enhance mosquito permissiveness to arboviruses [84,85]. In contrast, our recent investigation of *Serratia* strains isolated from *Anopheles* gut did not yield a significant impact on viral infection in *Aedes* mosquitoes, suggesting that these effects are likely strain specific. While most gut bacteria appear to have minimal impact on vector fitness or vector’s capacity to support pathogen development, a few bacterial strains from *Aedes* mosquitoes exhibit pronounced effects. One example is *Chromobacterium* sp., a non-symbiotic environmental bacterium with entomopathogenic properties. When introduced into mosquitoes via sugar meal, this bacterium was shown to inhibit both malaria parasites and dengue viruses in mosquito vectors, while also significantly reducing mosquito survival [86]. Its mosquitocidal effects underscore its potential as a biocontrol agent rather than a mutualistic symbiont. Another strain, *Chromobacterium* Csp_BJ, was found to secrete the lipase CbAEs, which lyses viral envelopes [87]. Notably, our recent work identified a novel anti-*Plasmodium* lipase from *Serratia ureilytica* Su_YN1 [41]. *Rosenbergiella*_YN46, isolated from field-collected *Aedes* mosquitoes, was shown to suppress flavivirus transmission by acidifying the mosquito midgut. This effect is mediated by a secreted glucose dehydrogenase, RyGDH, which inhibits viral infection of midgut epithelial cells [45].

### 3.3. Paratransgenesis or natural bacteria?

Both paratransgenesis and natural bacteria-based strategies offer unique advantages and face distinct challenges in MBD control. Paratransgenesis leverages synthetic biology tools to engineer bacteria capable of producing multiple pathogen-targeting effector molecules, providing flexibility and potential for targeting various pathogens simultaneously. This strategy also allows fine-tuning of effector expression and delivery, which can enhance efficacy and minimize potential ecological concerns or reduce fitness impacts on the host mosquito. In contrast, naturally occurring bacterial strains inherently colonize mosquito vectors and are typically viewed more favorably from a regulatory and public acceptance perspective, as they do not involve exogenous genetic modification. However, many of these gut bacteria exert their effects through poorly understood bioactive molecules and mechanisms, and their pathogen-blocking effects may be less robust than those achieved through engineered approaches.

Biosafety and regulatory considerations remain important for both strategies. Key concerns include off-target effects, unintended interactions with non-target organisms, and the need for rigorous safety evaluation—particularly in the case of engineered strains. Regulatory approval for paratransgenesis has historically been more complex due to the inclusion of synthetic constructs and potential gene flow elements such as antibiotic resistance cassettes. Nevertheless, the use of native symbiotic bacteria as chassis organisms and the development of self-limiting regulatory systems (e.g., blood-meal-inducible expression of effectors) [75] offer promising avenues to enhance biosafety and facilitate regulatory approval.

Despite their differences, both paratransgenic and natural bacteria-based approaches share key priorities: enhancing bacterial colonization and competitiveness in wild mosquito populations, developing standardized delivery and application methods, and navigating regulatory frameworks. Importantly, systematic exploration of field-collected mosquitoes for novel bacterial strains and bioactive molecules can inform the development of paratransgenic tools, while innovations in genetic engineering and containment strategies can also improve the safety and monitoring protocols that may also benefit native-strain approaches. Ultimately, it is important to emphasize that neither paratransgenesis nor natural bacteria approach alone is likely to achieve sustained reductions in MBD transmission. Instead, gut bacteria-based approaches must be integrated with existing and emerging vector control tools to maximize effect. For example, the combination of gut bacteria-based approach with chemical insecticides can enhance MBD transmission blocking in the remnant mosquito population. Similarly, combining gut bacteria-based approach with genetically modified mosquitoes could generate a synergistic effect, further reducing vector competence and accelerating progress toward disease elimination.

## 4. A Promising yet thorny road ahead

There are more than 3,000 mosquito species worldwide, yet only a small subset of mosquito species (most notably within the *Anopheles*, *Aedes*, and *Culex* genera) serves as major disease vectors. Conventional efforts to suppress or eliminate mosquito populations often prove unsustainable, raise substantial ecological concerns, and demonstrate diminishing effectiveness over time. A more sustainable long-term approach embraces the principles of the One Health framework, which emphasizes the interconnectedness of human health, animal welfare, and environmental integrity. Rather than killing mosquitoes, an alternative strategy seeks to modify vector populations to render them resistant to pathogen infection and, therefore, incapable of transmitting the pathogen to humans. This strategy includes transgenesis, in which mosquitoes are genetically engineered to become refractory to infection, as well as paratransgenesis or a symbiont-based approach, which involves introducing anti-pathogen gut symbiotic bacteria into mosquito populations to inhibit pathogen development and transmission. Such a “refractory” approach highlights the potential of vector-targeted interventions that interfere with pathogen development within the insect host, thereby blocking disease transmission without the need to eliminate mosquito populations (Table 2).

**Table 2:** Key characteristics of “kill” and “refractory” strategies in mosquito-borne disease control.

Strategy type	Kill strategy	Refractory strategy
**Primary objective**	Reduce mosquito population	Replace mosquito populations with individuals refractory to pathogen infection
**Implementation approaches**	Chemical insecticides, bioinsecticides, and sterile insect technique	Symbiont-based control, genetic modification
**Ecological impact**	Effects on non-target organisms, environmental contamination	Relatively limited ecological disruption
**Long-term efficacy**	Requires continuous application, high operational costs	Limited implementation with sustained efficacy (e.g., symbiont-based, paratransgenesis, or transgenesis strategies)

### 4.1. Symbiotic approaches in addressing emerging challenges

Symbiont-based control approaches have several advantages compared with other strategies. Certain mosquito-associated bacterial species—such as *Serratia* spp., *Asaia* spp., and other bacteria species—are well-adapted to colonize the mosquito midgut. These bacteria can proliferate after blood meals, when the mosquito acquires pathogens, and colonize multiple mosquito species. These properties support the potential for persistent pathogen-blocking effects in various mosquito populations. When deployed via sugar baits or other delivery methods, symbiont-based approaches can be particularly effective in targeting outdoor-biting mosquitoes, a major limitation of bed nets and indoor spraying. Importantly, paratransgenic approaches—engineered symbionts expressing anti-pathogen effectors—can be rapidly iterated and tailored to respond to emerging threats such as concurrent outbreaks of malaria and arboviruses. Moreover, symbiotic approaches are also inherently low-tech, low-cost, and require less human behavioral compliance, thus, they can be easily scaled up and widely implemented in underdeveloped countries. This is especially valuable in situations of reduced official commitment or funding and can easily be restored in cases of interruptions due to pandemics or extreme climate events.

Moreover, symbiont-based approaches are highly compatible with both established and novel vector control measures. They can be integrated with insecticide-impregnated bed nets, entomopathogenic fungi [88], and gene drive platforms to construct multifaceted intervention packages. Such combinations can create synergistic effects—lowering the threshold for epidemiological impact, mitigating resistance evolution, and accelerating progress toward sustained disease control and eventual elimination.

### 4.2. Mechanistic gaps in gut microbiota–pathogen–mosquito interactions

A deeper understanding of the mechanisms underlying symbiotic control approaches is crucial not only for enhancing their efficacy but also for gaining public and regulatory acceptance. Although promising, current research remains at an early stage. To optimize implementation, it is important to consider microbial ecological dynamics, including potential interactions between introduced bacteria and local microbiota. Given the genomic variability observed among bacterial strains—even within the same species—strain-level characterization is recommended to ensure consistency, functionality, and safety. This includes assessing traits, such as colonization capacity, genetic stability, and resistance profiles, which can influence performance and field outcomes.

The mosquito midgut, as the initial site of mosquito-borne pathogen development, serves as a complex interface, where host blood factors, mosquito-derived factors, and microbiota interact. These multipartite interactions collectively shape the mosquito’s vector competence, yet many of their underlying mechanisms remain unresolved. Recent studies have begun to reveal the intricacy of these interactions. For instance, host serum iron levels have been shown to modulate dengue virus acquisition by mosquitoes [89]. Additionally, tryptophan catabolism by gut bacteria influences gut barrier integrity and impacts *Plasmodium* infection susceptibility [90]. Furthermore, exposure to host blood serum induces symbiotic *Serratia* bacteria to produce outer membrane vesicles, which deliver effector proteins, such as lipases, that actively lyse *Plasmodium* parasites [83]. These findings underscore the importance of deciphering the complex interactions occurring within the mosquito gut, not only to clarify how symbiotic bacteria influence pathogen transmission but also to inform the development of next-generation MBD control strategies. A precise mechanistic understanding will be essential for optimizing and refining symbiotic control approaches, ensuring their long-term effectiveness and safe deployment in real-world applications.

### 4.3. Challenges limiting the implementation of symbiotic control approaches

Over the past two decades, symbiotic control strategies have demonstrated promising efficacy in laboratory setting and, more recently, in semi-field trials, supporting their potential in advancing MBD control. Despite these promising developments, the field now stands at a crucial crossroad. Key challenges include scaling up beyond pilot studies, fostering support among the public, policymakers, and regulatory bodies, defining professional and industrial standards, and translating research findings into commercially ready products. Although obstacles such as technical complexities, biosafety concerns, regulatory requirements, and funding limitations remain, the substantial benefits of these approaches justify continued commitment and investment.

Transitioning from research promise to practical implementation requires overcoming several interconnected barriers. Technically, scaling up production and formulation of viable symbionts necessitates robust quality-control systems, optimized delivery platforms, and validated assays to reliably measure pathogen-blocking efficacy under diverse field conditions. Safety and ecological assessments—including evaluations of horizontal gene transfer, non-target organism impacts, and evolutionary stability—must conform to rigorous international guidelines. Furthermore, transparent engagement with local communities, clear communication of risks and benefits, and equitable financial support mechanisms are essential to securing public acceptance and regulatory approval. With coordinated progress across these domains, symbiotic control approaches are well-positioned to move beyond the experimental stage and emerge as transformative, scalable tools in the global fight against MBDs.

## 5. Concluding remarks

### 5.1. Bridging the gap: From laboratory to semi-field and field trials

Recent advances in symbiotic control strategies have underscored the critical importance of semi-field testing as an essential step toward real-world application. These trials serve as a vital bridge between laboratory studies and full-scale field deployment, helping to evaluate the effectiveness, identify potential impacting factors, and refine methodologies before large-scale implementation. While paratransgenesis still faces regulatory resistance in transitioning to semi-field and field studies, natural symbiotic gut bacteria-based approaches have already seen significant progress in this area. Several symbiotic bacteria with pathogen-blocking properties, such as Su_YN1 and TC1 (both anti-*Plasmodium*) and YN_46 (anti-arbovirus) [41,44,45], demonstrate strong feasibility for larger-scale semi-field studies and even early-stage field trials. However, such real-world testing requires conducting experiments in authentic disease-endemic settings, often entailing extensive international collaboration, complex regulatory approvals, long experimental timelines, and substantial financial and logistical investment. Additionally, these trials are subject to seasonal and climatic variations, as well as potential political instability in test regions, further complicating their execution. Given these challenges, the successful advancement of semi-field and field trials will require strong governmental cooperation and the support of international organizations such as the World Health Organization (WHO).

### 5.2. Field deployment strategies

Efficient deployment of pathogen-blocking bacteria into wild mosquito populations is critical to the successful implementation of symbiotic control strategies. Among various options, sugar-bait stations containing symbiotic bacteria represent the most practical and scalable delivery method. These stations capitalize on the natural sugar-feeding behavior of both male and female mosquitoes, require no mass-rearing infrastructure, and can be flexibly distributed throughout villages, peri-domestic vegetation, or livestock shelters. Once established, community health workers can easily service these stations at relatively low operational costs. Initial field trials have demonstrated that high symbiont prevalence can be maintained, even within highly mobile vector populations.

Importantly, sugar-bait stations alleviate public concerns associated with large-scale mosquito releases and significantly reduce labor demands, making this approach particularly suitable for deployment in resource-limited regions such as sub-Saharan Africa. By comparison, direct inoculation of larval habitats, although conceptually appealing, faces substantial operational difficulties due to the abundance, transient nature, and seasonal variability of breeding sites, especially after rain events. Similarly, releasing laboratory-reared, symbiont-infected adult mosquitoes remains the most resource-intensive and operationally complex strategy, requiring extensive insectary capacity, sex separation, stringent quality controls, specialized transportation, and rigorous regulatory oversight.

Sugar-bait stations are already widely utilized for field-based mosquito assessments across Africa [91], demonstrating their immediate practicality and community acceptance as bacterial delivery systems. Nevertheless, technological refinements remain necessary. A recent field trial of attractive targeted sugar baits for malaria control in western Kenya showed no reduction in mosquito density or malaria endpoints [92], perhaps owing to insufficient lure power of the baits and low effective coverage of the device. Clay pot combined with compound fruit juice baits [93], offers a technology that is close to the feeding and resting behavior of mosquitoes, and provides a feasible way in disseminating symbiotic bacteria. For optimal efficacy, bait attractants must be tailored to local mosquito feeding preferences, and bacterial formulations must ensure prolonged viability under field conditions, including exposure to heat and UV radiation. In addition, contamination-prevention measures are needed to protect bait formulations from environmental microbes.

### 5.3. Remaining challenges and the road ahead

**Fig 5:**
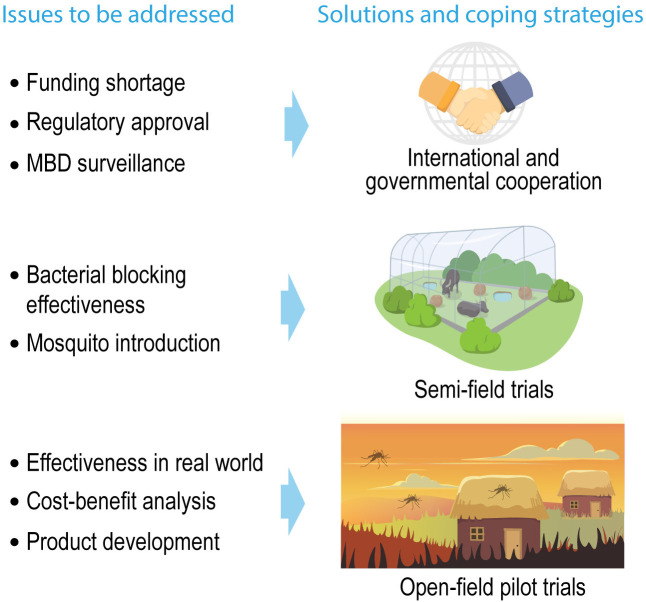
Symbiotic control strategies–opportunities and challenges. Key issues need to be addressed in translating symbiotic control strategies in the field.

Despite significant advancements in symbiont-based transmission-blocking strategies, numerous scientific, technical, and regulatory challenges remain before these approaches can be widely implemented (Fig 5). Key hurdles include improving the ability of bacteria colonization of mosquitoes, improving competitiveness of engineered or natural bacteria in wild mosquito populations, optimizing large-scale deployment methods, and ensuring the long-term stability of these interventions in diverse environmental conditions. Furthermore, regulatory approval and public acceptance are essential and pose major challenges, particularly for paratransgenesis, as concerns over biosafety, ecological impact, and off-target effects must be rigorously addressed. Even natural bacterial approaches require comprehensive validation to confirm their efficacy and safety in real-world applications. At the same time, the evolving epidemiology of MBDs—driven by climate change, insecticide resistance, urbanization, and global human mobility—demands constant innovation. Importantly, the interplay between “kill” and “refractory” strategies must be considered. Integrating symbiotic control with existing tools such as insecticide-based approaches, genetic vector control, and public health interventions is a must to create holistic solutions.

Achieving sustainable MBD control will require long-term investment, interdisciplinary collaboration, and international cooperation. While the path ahead is filled with challenges, the introduction of symbiotic control technologies—guided by fundamental research and field-based evidence—holds potential to transform the future of vector-borne disease suppression.
